# Can detection and prediction models for Alzheimer’s Disease be applied to Prodromal Parkinson’s Disease using explainable artificial intelligence? A brief report on Digital Neuro Signatures.

**DOI:** 10.12688/openreseurope.14216.2

**Published:** 2022-01-10

**Authors:** Ioannis Tarnanas, Panagiotis Vlamos, Dr Robbert Harms, The RADAR-AD Consortium

**Affiliations:** 1 Altoida Inc., Washington DC, Washington, DC (DC), 20003, USA; 2 Bioinformatics and Human Electrophysiology Laboratory (BiHELab), Department of Informatics, Ionian University, 7 Tsirigoti Square, Corfu, Greece

**Keywords:** prediagnostic Parkinson's disease, digital neuro signatures, prodromal Parkinson's disease

## Abstract

Parkinson's disease (PD) is the fastest growing neurodegeneration and has a prediagnostic phase with a lot of challenges to identify clinical and laboratory biomarkers for those in the earliest stages or those 'at risk'. Despite the current research effort, further progress in this field hinges on the more effective application of digital biomarker and artificial intelligence applications at the prediagnostic stages of PD. It is of the highest importance to stratify such prediagnostic subjects that seem to have the most neuroprotective benefit from drugs. However, current initiatives to identify individuals at risk or in the earliest stages that might be candidates for future clinical trials are still challenging due to the limited accuracy and explainability of existing prediagnostic detection and progression prediction solutions. In this brief paper, we report on a novel digital neuro signature (DNS) for prodromal-PD based on selected digital biomarkers previously discovered on preclinical Alzheimer's disease. (AD). Our preliminary results demonstrated a standard DNS signature for both preclinical AD and prodromal PD, containing a ranked selection of features. This novel DNS signature was rapidly repurposed out of 793 digital biomarker features and selected the top 20 digital biomarkers that are predictive and could detect both the biological signature of preclinical AD and the biological mechanism of a-synucleinopathy in prodromal PD. The resulting model can provide physicians with a pool of patients potentially eligible for therapy and comes along with information about the importance of the digital biomarkers that are predictive, based on SHapley Additive exPlanations (SHAP). Similar initiatives could clarify the stage before and around diagnosis, enabling the field to push into unchartered territory at the earliest stages of the disease.

## Introduction

Parkinson's disease (PD) prevalence rate increases with age and rises from about 1% in individuals aged ≥60 years to 3.5% in older adults of 85–89 years ^1– 3^. The complexity of cross-sectional diagnosis is stereotypically exemplified in PD, which happens to be the second-most common neurodegenerative disorder after Alzheimer's disease ^4– 6^. The pathological hallmark of PD is misfolded α-synuclein protein (aSyn) structures, and the gold standard for diagnosis is their identification in *post mortem* pathological examinations of the brain ^7^. However, given that most idiopathic patients experience years or sometimes decades of unspecific symptomology, the field of prediagnostic Parkinson's disease (prodromal-PD) is fast-moving with multiple strategies seeking to discover a panel of clinical and laboratory biomarkers for those 'at risk' ^8^. Prodromal-PD ^9^ is when individuals do not fulfill diagnostic criteria for PD ( *i.e*., bradykinesia and at least one other motor sign) but exhibit signs and symptoms that indicate a higher-than-average risk of developing motor symptoms and a diagnosis of PD in the future. Presently, most imaging markers across a range of modalities and the emerging literature on fluid and peripheral tissue biomarkers is limited in predicting prodromal-PD, pointing to the need to identify robust predictors of change across the entire spectrum from ordinary to symptomatic PD for more realistic primary or secondary preventive trials for PD ^10^.

Consequently, longitudinal measures of pre-motor symptoms and behavioral/cognitive decline are essential for evaluating preclinical markers and monitoring prodromal-PD progression. Such longitudinal characterization of non-motor features has been identified by the Movement Disorders Society (MDS) as being valuable for early identification of PD, according to the research criteria for prodromal PD ^11^, which include two types of measurements: the delineation of the relative temporal trajectories of specific quiet motor and non-motor features that can be present before diagnosis and the fluctuation of those features over time within and across neurocognitive domains ^12^. The utility of such markers in evaluating prodromal-PD progression depends on early symptoms and signs before PD diagnosis is possible and may vary across different primary care settings ^12^. The utility of such markers in evaluating prodromal-PD progression depends on early symptoms and signs before PD diagnosis is possible and may vary across different primary care settings ^12^. However, intra-individual variability (IIV) across several measurements, called dispersion, is a sensitive marker for detecting change even at prodromal stages of a disease ^13^. One digital biomarker tool that utilizes dispersion to provide such measurements is the Altoida Digital Neuro Signatures platform (DNS), a more efficient, accurate, and sensitive assessment of cognitive function than traditional neuropsychological tests, both in cross-sectional and longitudinal evaluations ^14^. Previous studies have validated the machine learning model's performance to measure dementia disease progression and detect the biological signature of prodromal AD, which predicts conversion from mild cognitive impairment (MCI) to Alzheimer's disease (AD) with 94% prognostic accuracy ^15^.

In this work, we will briefly report on DNS signature similarities from previous studies and the dataset collected in The ANANEOS Project, an ambitious longitudinal community-based study for healthy aging in Greece. The project is part of the GR2021 Priority project Healthy Brains for Life (age 20–99 years) and focuses on the decentralized and remote assessment of the symptoms of preclinical stages in Alzheimer's disease and movement disorders, *e.g*., Parkinson's, with a rationale and a methodology similar to other international initiatives. Relevant examples of similar large-scale national initiatives can be found in Japan with the IROOP registry system for identifying risk factors for dementia ^16^, the Sydney (Australia) memory and ageing study ^17^, the Framingham heart study in the USA ^18^, the UK Biobank study of lifestyle and genetic factors incidence in dementia ^19^, the European Prevention of Alzheimer’s Dementia Longitudinal Cohort Study ^20^, the FINGER project in Finland ^21^, the INTERCEPTOR Project in Italy ^22^ or The Vallecas Project in Spain ^23^.

The emergence of large longitudinal primary care cohorts, alongside advances in digital biomarkers and artificial intelligence (AI), has allowed detailed exploration of the full range of early motor and non-motor symptoms that predate PD. In contrast, advanced prodromal PD detection and prediction models could become a platform for medical practitioners that plan to diagnose or detect the disease earlier and more accurately. Despite the enthusiasm that objective motor dysfunction occurs prior to diagnosis in PD and the variety of measuring devices, which have been developed, including software applications that harness passive and active digital biomarkers, *e.g*. activity and motion (and in some cases speech) captured by smartphones and tablet devices, custom-built sensors that measure gait, bradykinesia, dyskinesia, and nocturnal movement detection devices, there are currently very few examples of the application of wearable devices or AI models in prediagnostic PD.

Our goal here was to answer the single question: Can detection and prediction models for Alzheimer's disease be rapidly applied to prodromal Parkinson's disease using explainable artificial intelligence? A major foreseeable hurdle is ensuring that any detection and prediction model focuses both on improving the system performance and AI interpretability, employing natural language explanations, which could help physicians understand the predictions. For the answer above, we focused on DNS signature patterns between our existing databases and ANANEOS using permutation-based techniques to help us understand the actual effect of the predictors (DNS signatures from the existing AD database) in the target database (preclinical markers that predict Prodromal-PD progression).

## Methods

### Data collection

We used a combination of clinical and population data, collected and provided by Altoida, Inc. The clinical data (n=438) is described in previous studies ^15^ and consists of controlled tests of elderly (≥50 years) subjects with known biological and psychological biomarkers ( *e.g*., MCI, amyloid-beta (Ab)+, Ab-, AD). We used the dataset described as "New validation study" (ClinicalTrials.gov Identifier: NCT02843529) for this work, the original purpose of which was to evaluate the performance of Altoida's application as an adjunctive tool for diagnosing AD. This data was collected in various major cities in Italy, Greece, Spain, USA, and Ireland.

The dataset above was enriched with two more databases:

1) A clinical dataset called RADAR-AD. RADAR-AD is a multicentre observational, cross-sectional, cohort study in subjects within the preclinical-to-moderate AD spectrum as well as healthy controls. The design entails three tiers: (1) main study, which includes smartphone applications and wearable devices only; (2) first sub-study, which in addition includes fixed sensors at the participant’s home; and (3) second sub-study, which in addition includes fixed sensors in an existing smart home environment. Participating clinical sites were selected based on their geographic location, expertise in digital technologies and disease population of interest, and the availability of clinical cohorts with known AD biomarkers ^24^.
2) A population dataset collected by Altoida named "healthy basket." A healthy basket is a population sample consisting of middle-aged cognitively healthy Japanese subjects (n=130). The inclusion criteria for participation were age 20–50 years and self-assessed cognitively healthy ( *i.e*., no known cognitive disorders). The subjects received no stipend for participation, and permission for scientific studies was provided by accepting the terms and conditions of Altoida, Inc. All subject information was anonymized and de-identified. Beyond the digital biomarkers collected by the Altoida application, no further biomarkers were recorded for this population sample. For both datasets, the subject's sex was self-reported. All subjects (of both groups) performed multiple test sessions using Altoida's application.

Finally, the target database was part of the Digitally enhanced, Decentralized, Multi-omics Observational Cohort (ANANEOS) study. ANANEOS is an ongoing single-centre, observational, longitudinal cohort (n=500,000) for individuals (aged ≥50 years) with a ClinicalTrials.gov Identifier: NCT04701177. The participants, recruited initially since March 2021 in Athens, Greece, are home-dwelling volunteers with known biological and psychological biomarkers at the preclinical stages in Alzheimer's disease and movement disorders, *e.g*., Parkinson's, without relevant psychiatric, neurological, or systemic disorders. The initial cohort size was 2,180 subjects at baseline. At the time of this writing (10/13/2021), the project is in the first wave of the 24-week follow-up visits (n=133).

**Figure 1.**
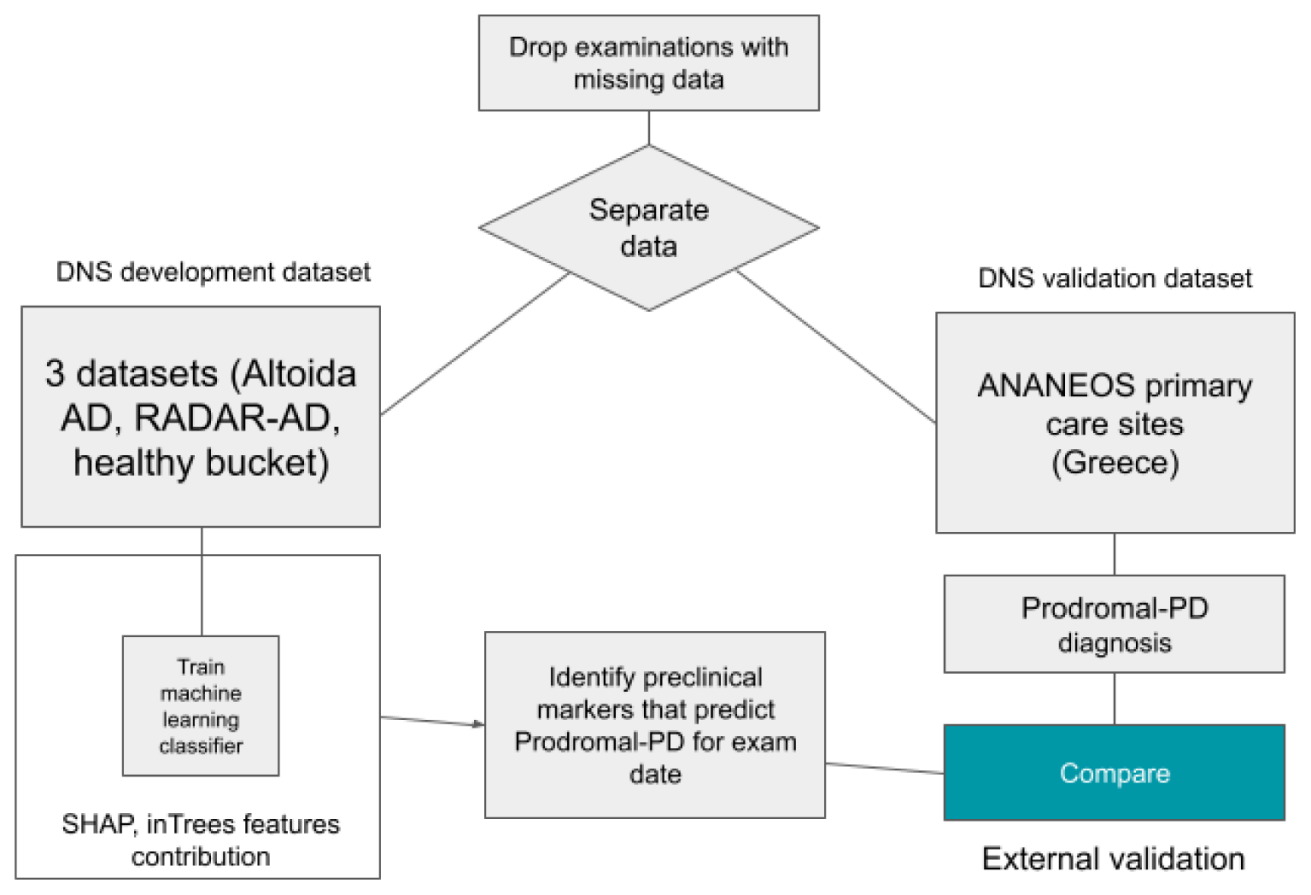
Flowchart showing the overall dataset structure and the prelim study purpose.

Table 1 describes our data characteristics for the entire sample and stratified by sex, with univariate comparisons. Our data consists of 788 subjects combined from three datasets, two clinical datasets ^15, 24^ and a healthy population dataset. Subjects were distributed over several stages of the AD clinical continuum, namely healthy, preclinical AD Ab+, MCI (amyloid-βnegative) Ab-, MCI Ab+, dementia due to AD and prodromal PD as reported by clinical assessment. To counter the imbalance from multiple data points per subject and combining two demographically different datasets, we stratified all analysis by dataset, sex, and number of data points. This ensures that we have exactly the same number of data points from each sex and from each study (clinical and population). The flowchart showing the overall dataset structure and the prelim study purpose is shown in Figure 1.

**Table 1.** Data characteristics. P-value is calculated using a two-sided t-test for age, chi2 for status and the Mann-Whitney rank test for the number of data points per subject.

			Men	Women	Total	p-value
Population	Clinical data	N (%)	280 (42%)	378 (58%)	658	1.2e-12
	Population data	N (%)	94 (72%)	36 (28%)	130	
	ANANEOS	N (%)	66 (50%)	67 (50%)	133	
Age		Mean (SD)	56.9 (17.4)	62.7 (12.8)	60.1	7.5e-06
Status	Healthy	N (%)	198 (45%)	237 (55%)	435	0.786
	Preclinical AD	N (%)	103 (46%)	117 (54%)	220	
	MCI ab-	N (%)	16 (38%)	26 (62%)	42	
	MCI ab+	N (%)	35 (44%)	43 (56%)	78	
	AD	N (%)	5 (38%)	8 (62%)	13	
	Prodromal PD	N (%)	13 (54%)	11 (46%)	24	
Number of DNS trials (data points)		N (%)	1448 (52%)	1359 (48%)	2807	-
Number of DNS trials (data points) per subject		Median (IQR)	2 (4)	2 (5)	2 (3)	2.8e-05

### Digital neuro signatures (DNS)

**Figure 2.**
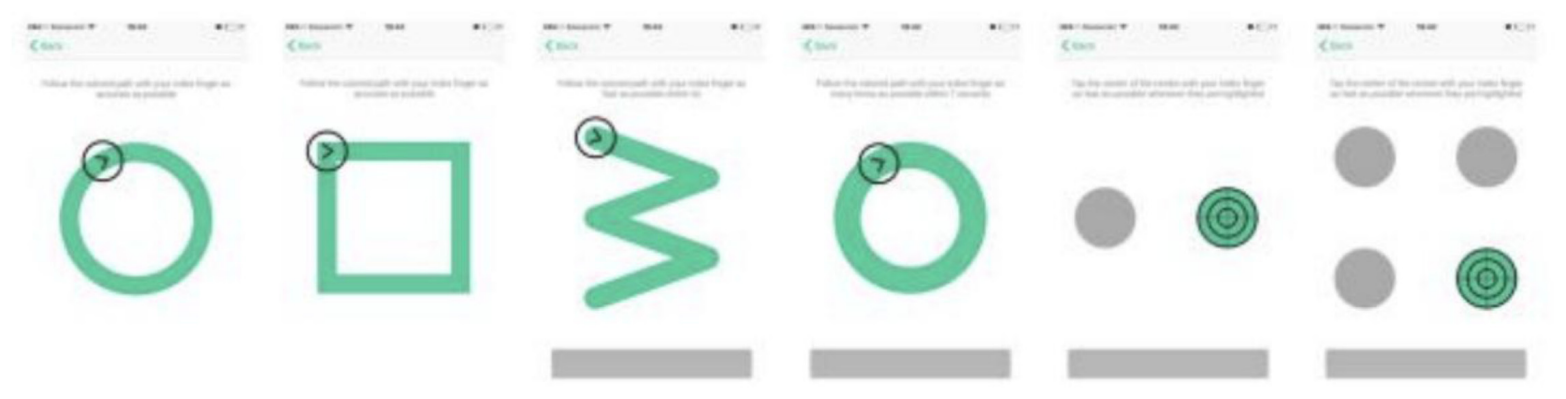
The motoric functioning tasks in the Altoida test. These are executed one after another. Using their index finger of their dominant hand, from left to right, the task is to 1) draw a circle, 2) draw a square, 3) draw a rotated W shape within 7 seconds, 4) draw as many circles as possible within 7 seconds, 5) tap the highlighted buttons (left, right, left, right, etc.) 6) tap the highlighted button as fast as possible, the buttons highlight at random.

**Figure 3.**
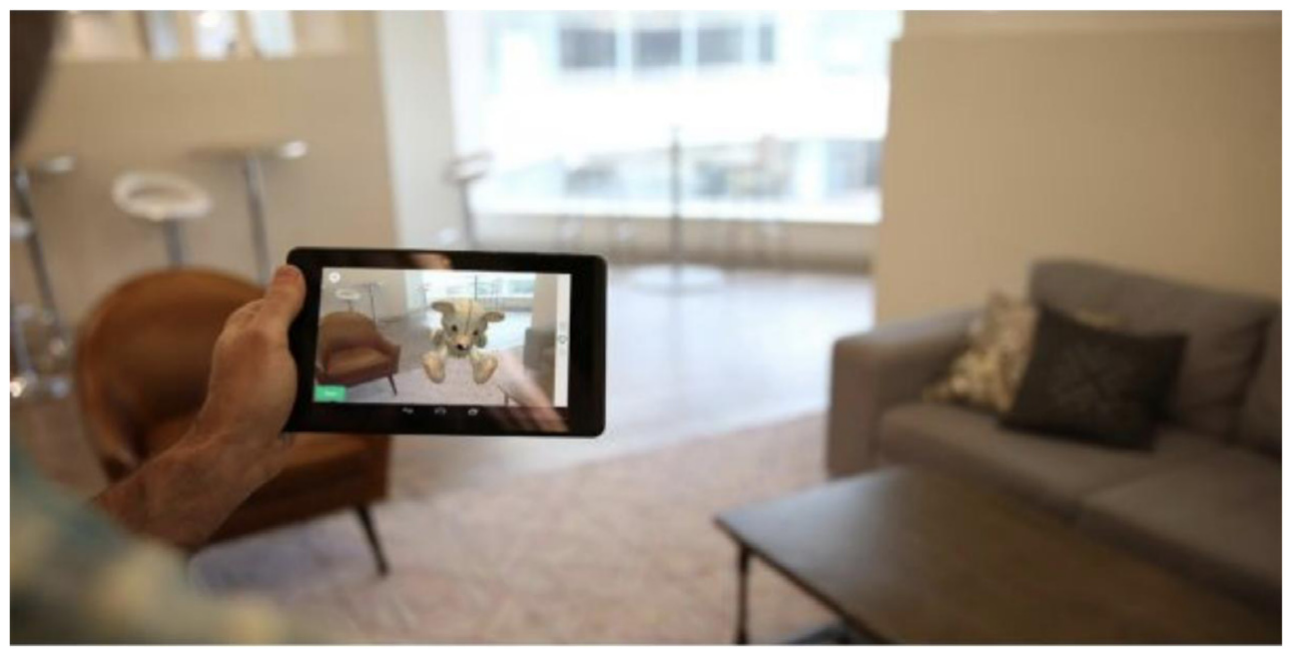
Illustration of the Augmented Reality (AR) task in the Altoida test. During the AR test, the subject is asked to place and find three virtual objects in the room. To do so, the subject is required to walk around the room holding a tablet or smartphone device in front of him/her. While doing so, the camera of the device records the environment and displays it back to the user on the screen, augmented with virtual objects (in this illustration, a teddy bear). The user needs to place the objects on flat surfaces and later recall their position by walking back to that location.

For this work, we repurposed data from Altoida’s application which collects digital biomarkers for neurocognitive function measurement and predictive diagnosis of AD ^15^. Altoida's application collects digital biomarker data for detecting early-onset AD. While holding a tablet or smartphone device, the subject is asked to perform a series of motor functioning tasks and two augmented reality (AR) tasks. In the motor functioning tasks, the subject is required to draw shapes and tap on the (touch)screen using the finger of their dominant hand (see Figure 2 for an illustration of all the motor functioning tasks). In one of the AR tasks, the subject is asked to place three virtual objects in a small space (approximately 3×3 m or 2×4 m) and afterward find them again. The AR task is performed by navigating around the space with the tablet or smartphone in both hands (see Figure 3). During these tasks, the handheld device collects telemetry and touch data from the built-in sensors, enabling profiling of hand micro-movements, screen-touch pressures, walking speed, navigation trajectory, cognitive processing speed, and additional proprietary inputs.

A single test session using Altoida’s application consists of two batches of motor tasks and two AR tasks. After a subject completes all tasks, the recorded digital biomarker data from the onboard electronics sensors is bundled and securely and anonymously uploaded to a server for further processing. Provided the data of multiple subjects, machine learning can be used to detect patterns. In previous work machine learning was either used to classify subjects as healthy or at risk of AD ^15^. In this work, we examined DNS signatures from our development dataset for AD to see if they demonstrated preclinical markers that predict prodromal-PD progression, expressed by the capacity of results to inform a novel prodromal-PD DNS signature.

### Machine learning

We extracted 793 digital biomarker features from the onboard electronics sensors describing various cognitive, functional, and physiological characteristics of each subject. These features include response times, eye-hand coordination precision, fluctuations in the telemetry (accelerometer and gyroscope) data, Fourier analysis of the telemetry data, step detection, and additional proprietary data. Based on the digital biomarker feature data from a selection of healthy subjects, we trained a DNS-match classifier to distinguish prodromal-PD individuals from any other group. We used the XGBoost algorithm ^25^ with DNS preclinical markers that predict prodromal-PD as the target variable for the classification.

### Performance evaluation

We applied stratified five-fold grouped cross-validation to estimate the generalization performance of the DNS prodromal-PD classifier. We grouped data points by subject to ensure that multiple data points of a single subject were all in the same fold (either training or testing), preventing learning bias. For our prodromal-PD classifier, we measured accuracy and precision averaged over the five cross-validation testing folds. To assess the classifier's performance on different age groups, we trained nine additional classifiers (10 in total), each using different random subsets of the data.

### Model explainability

We used the Shapley Additive exPlanations (SHAP) ^26^ method to better understand the predictions made by the DNS prodromal-PD classifier. The SHAP method allocates to each feature of a classifier a game-theoretical value representing the contribution of that feature towards the classification targets. The sign of the SHAP values indicates the direction of the contribution, and the magnitude of the SHAP value indicates the importance. For our classifier, negative SHAP values contribute to classifying as non-prodromal-PD, positive numbers towards prodromal-PD. SHAP values have an additive property meaning they can be summed together to provide the feature contribution of a group of features ^27^.

## Results

### Common DNS signature for preclinical AD and prodromal PD and features contribution

**Figure 4.**
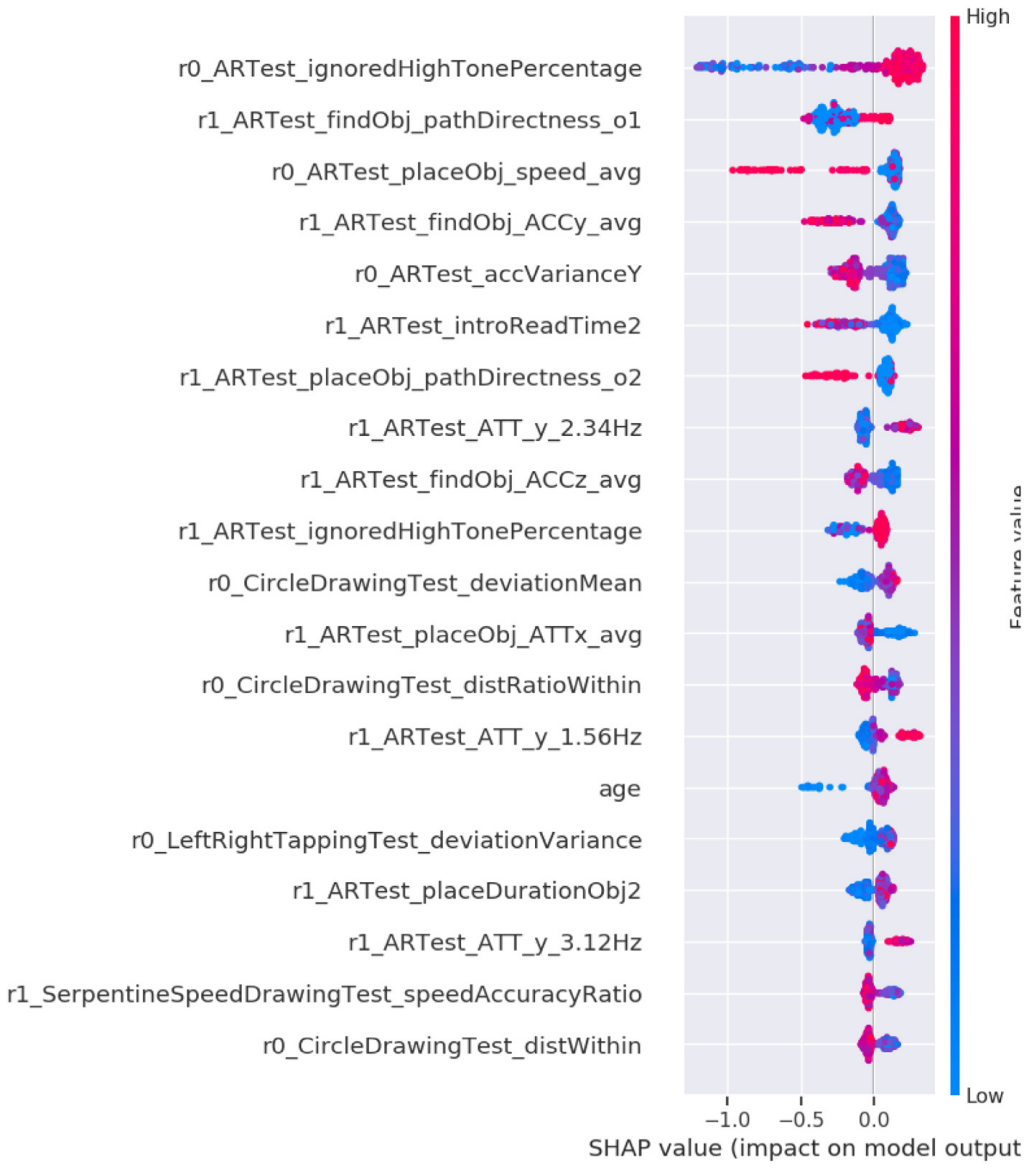
Feature importance of the Prodromal-PD classifier. **A**) The top twenty feature groups according to the SHAP method. Each bar represents the summed SHAP value of the features in that feature group. **B**) A feature value SHAP distribution plot for the top five contributing features. Subject specific SHAP values were computed for each datapoint in the classifier training data. For each feature, we then plot for each datapoint a dot with the feature value of that datapoint, with the dot color coded by the relative feature value. The position of each dot on the SHAP value x-axis represents the magnitude and the direction of the contribution of that specific feature value of that specific datapoint towards classifying as female (-1) or male (+1). Acronyms in the plots are Augmented Reality (AR), Fast Fourier Transform (FFT), SHapley Additive exPlanations (SHAP), Accelerometer (ACC), variance (var), first part of a single test (1st) or second part of a single test (2nd).

We wanted to investigate whether detection and prediction models for Alzheimer's Disease can be rapidly applied to prodromal PD using explainable artificial intelligence. The analysis of our prodromal PD classifier revealed at least 20 common features that are the same for both preclinical AD and prodromal PD. After computing a SHAP value for each DNS signature containing more than 793 features from our development dataset, we arrived at this conclusion. We compared them with a novel prodromal-PD classifier, which detected a DNS signature in the ANANEOS validation dataset. We then investigated, which of the 793 features contain the most relevant preclinical markers that predict prodromal-PD. Figure 4 shows a grouping of those features that were ranked as having the highest overall contribution in the classifier.

The primary contributing group of features is named the ignore high tone percentage and the AR object placement directness. This group consists of an interference index (non-motor feature) and a set of frequency magnitudes obtained while the participant moves around trying to find a virtual object in the AR test (motor feature). These features could therefore be interpreted as a brain network function and navigation micro-errors. The second most important group of digital biomarker features is the AR global telemetry variance. The global telemetry variance is the variance in the accelerometer and gyroscope signal over the entire duration of the AR task. It could be interpreted as coarse-scale hand motion micro-movement (motor feature). The third and fourth most essential features are frequency magnitudes during object placement, belonging to the top group placing virtual objects in the AR test, collected using a fast Fourier transform (FFT) on the measured accelerometer and gyroscope signal over 1.28 seconds before placing (motor feature). The remaining elements of the novel DNS prodromal-PD signature are taking into account age and group together "Motor test drawing features" to consider the speed and accuracy of the subject while drawing various patterns with the index finger (motor feature). The "Circle drawing test" measures how long the user spent within the limits of the circle while performing the motor tests. 

## Conclusion

Our work demonstrates that it is possible to detect a novel DNS signature from existing datasets using digital biomarker data collected from Altoida's application. The intrinsic similarities between preclinical AD markers and preclinical markers that predict prodromal-PD seem to be capturing quiet motor and non-motor features dependent on age. In the prediagnostic Parkinson's Disease population, the primary differentiating features are micro-errors and micro-movements detectable by Fourier analysis on accelerometer data, although they are non-visible to the naked eye. Such prelim results can provide physicians with some insights into driving factors of our prediction model from multiple points of view including visualization, and feature importance based on SHapley Additive exPlanations (SHAP). Further validation is pending upon larger sample size and multiple additional biological markers and endpoints.

## Data availability

The data that support the findings of this study are available from Altoida Inc., but restrictions apply to the availability of these data, which were used under license for the current study, and so are not publicly available. Data are however available from the authors upon reasonable request and with permission of the RADAR-AD consortium.

## Disclaimer

This communication reflects the views of the RADAR-AD consortium and neither IMI nor the European Union and EFPIA are liable for any use that may be made of the information contained herein.

## Link to IMI website

**www.imi.europa.eu**
