# Supplementary material for: Can detection and prediction models for Alzheimer’s Disease be applied to Prodromal Parkinson’s Disease using explainable artificial intelligence? A brief report on Digital Neuro Signatures
Source: Open Res Eur. 2022 Jan 10;1:146. Originally published 2021 Dec 3. [Version 2] doi: 10.12688/openreseurope.14216.2 (PMC10445877; doi:10.12688/openreseurope.14216.2)
Supplement: Peer review history

# Peer review: Reviewer response for version 2

**DOI:** 10.21956/openreseurope.15552.r28293

**Published:** 2022-01-10

**Authors:** Adria Tort

**Affiliations:** 1 Alzheimer's Disease and Other Cognitive Disorders Unit, Neurology Service, Hospital Clínic de Barcelona, Institut d'Investigacions Biomèdiques August Pi i Sunyer (IDIBAPS), University of Barcelona, Barcelona, Spain

I accept the addition of the new co-author.

Is the study design appropriate and does the work have academic merit?

Yes

Is the work clearly and accurately presented and does it cite the current literature?

Yes

If applicable, is the statistical analysis and its interpretation appropriate?

Yes

Are all the source data underlying the results available to ensure full reproducibility?

Yes

Are the conclusions drawn adequately supported by the results?

Yes

Are sufficient details of methods and analysis provided to allow replication by others?

Yes

Reviewer Expertise:

Neuropsychology, Alzheimer's disease, Dementia, Biomarkers, early detection

I confirm that I have read this submission and believe that I have an appropriate level of expertise to confirm that it is of an acceptable scientific standard.

# Peer review: Reviewer response for version 1

**DOI:** 10.21956/openreseurope.15331.r28136

**Published:** 2021-12-22

**Authors:** Adria Tort

**Affiliations:** 1 Alzheimer's Disease and Other Cognitive Disorders Unit, Neurology Service, Hospital Clínic de Barcelona, Institut d'Investigacions Biomèdiques August Pi i Sunyer (IDIBAPS), University of Barcelona, Barcelona, Spain

Tarnanas *et al.* present an interesting study on the application of Alzheimer’s disease detection and prediction models for the identification and monitoring of prodromal stages of Parkinson’s disease. The use of IA-driven assessments on these populations provides an alternative method for detecting subtle cognitive deficits preceding the symptomatic phases of Parkinson’s disease in a more sensitive way that the one provided by standard neuropsychological measures. The predictive value of the digital neuro signature (DNS) appears very promising. This is an interesting, well-justified and well-reported and analyzed large-scale study and deserves indexing.

 I would like to ask the authors some questions:

- Cognitive performance is strongly correlated with age. Also, the use of novel devices and techniques in older adults may be mediated by the participant’s skills and familiarization. How do the authors think this could impact task sensitivity and specificity? How easy-to-run is the Altoida’s application and how learning effects were controlled?
- The present findings are in line with previous studies suggesting that individuals within the preclinical phase of Alzheimer’s disease may exhibit subtle motor dysfunction (Albers *et al.*, 2015; Buchman & Bennett, 2011) and that intrasubject variability (i.e., the inconsistency of performance) might be a more relevant marker of early AD than motor speed (Verghese *et al.,* 2008; Mollica *et al.*, 2019). Also, another less-studied variable such as multitasking performance appears to be a sensitive indicator of subtle dysfunction. Notwithstanding the conclusion that the DNS seems to be a valid probe of early cognitive changes in at-risk populations, do the authors think that further research is needed to isolate the critical elements of presymptomatic evaluation?
- How do the authors think that their approach could be effectively implemented in both clinical (e.g., primary care) and research (e.g., clinical trials) settings?

Finally, I would like to congratulate the authors for this interesting report and encourage them on providing further insights for this potential digital biomarker.

Is the study design appropriate and does the work have academic merit?

Yes

Is the work clearly and accurately presented and does it cite the current literature?

Yes

If applicable, is the statistical analysis and its interpretation appropriate?

Yes

Are all the source data underlying the results available to ensure full reproducibility?

Yes

Are the conclusions drawn adequately supported by the results?

Yes

Are sufficient details of methods and analysis provided to allow replication by others?

Yes

Reviewer Expertise:

Neuropsychology, Alzheimer's disease, Dementia, Biomarkers, early detection

I confirm that I have read this submission and believe that I have an appropriate level of expertise to confirm that it is of an acceptable scientific standard.

## References

[1] : At the interface of sensory and motor dysfunctions and Alzheimer's disease. *Alzheimers Dement* .2015;11(1) : 10.1016/j.jalz.2014.04.514 70-98 10.1016/j.jalz.2014.04.514 25022540PMC4287457

[2] : Loss of motor function in preclinical Alzheimer's disease. *Expert Rev Neurother* .2011;11(5) : 10.1586/ern.11.57 665-76 10.1586/ern.11.57 21539487PMC3121966

[3] : Gait dysfunction in mild cognitive impairment syndromes. *J Am Geriatr Soc* .2008;56(7) : 10.1111/j.1532-5415.2008.01758.x 1244-51 10.1111/j.1532-5415.2008.01758.x 18482293PMC2574944

[4] : Early detection of subtle motor dysfunction in cognitively normal subjects with amyloid-β positivity. *Cortex* .121: 10.1016/j.cortex.2019.07.021 117-124 10.1016/j.cortex.2019.07.021 31561128

# Response

**Published:** 2021-12-22

**Authors:** Ioannis TARNANAS

Dear Dr. Tort, many thanks for your thorough review and excellent questions. We would like to address them here for your attention:

- Learning effects and the influence of tech literacy have indeed being measured through another study performed by an independent third party. Since this is a brief report for the specific finding about PD, we are reserving the right to publish those results at another publication.
- Similar to the answer above, yes further analysis of the psychometric properties of the test are also being computed. The clinical protocol of that study will be published soon. It has been submitted at another journal.
- The application of digital biomarkers in clinical practice and clinical trials is actually a very interesting question. Different barriers and opportunities exist in those two areas. For clinical trials, we aim to become a secondary/primary endpoint that could identify and report meaningful change of different groups or patients, e.g. Preclinical PD, Prodromal PD and PD diagnosis for therapeutic clinical trials. In the clinical domain and once the results of the primary endpoint above are satisfactory, we would like to become a companion diagnostic for novel treatments. In general however the barriers of entering clinical practice in the primary care setting is a multifaceted one. It involves a dialogue with regulators, medical associations, healthcare economics (HTA) and other units that spread a long way after the demonstration of the clinical benefits. Therefore, we do believe that the first to benefit are going to be the clinical trials and after a long time also the primary care setting.

# Peer review: Reviewer response for version 1

**DOI:** 10.21956/openreseurope.15331.r28143

**Published:** 2021-12-13

**Authors:** Renato Anghinah

**Affiliations:** 1 Department of Neurology, University of São Paulo, Sao Paulo, Brazil

1. First of all, I would like to congratulate the authors for the high level of this paper. AI and learning machines have been used to help MD take decisions about diagnosis and treatments in a wide range of diseases.
  A very important area that needs this kind of approach is cognitive disease. The computational solution used to Alzheimer’s Disease (AD) from Altoida device just showed useful and Digital Neuro Signature (DNS), was a good candidate to a new biomarker. The use of XGBoost algorithm is an excellent choice once in my point of view is the best machine learning to be used for structured data, like what we have in this study.
2. I have two questions for the authors: the first one is how they explain the fact that the same 20 features, in 793 possibilities,  are the best for AD and Parkinson’s disease (PD), when we know that in AD disease the cognitive impairments are more important, instead of in PD the motor ones are the main problem mainly in the beginning of these diseases?
  The second one is: If each one item of the 20 features is compared head to head are there differences between them, and in case of positive answer, what are they?
3. As explained above, is an excellent paper, well written, with a good methodology, and deserves to be published. My opinion is this paper is approved.    

Is the study design appropriate and does the work have academic merit?

Yes

Is the work clearly and accurately presented and does it cite the current literature?

Yes

If applicable, is the statistical analysis and its interpretation appropriate?

Yes

Are all the source data underlying the results available to ensure full reproducibility?

Yes

Are the conclusions drawn adequately supported by the results?

Yes

Are sufficient details of methods and analysis provided to allow replication by others?

Yes

Reviewer Expertise:

Neurology, cognitive and behavioral neurology ,trauma Brain Injury, Clinical Neurophisiology

I confirm that I have read this submission and believe that I have an appropriate level of expertise to confirm that it is of an acceptable scientific standard.
